# Responses of Nutrients and Mobile Carbohydrates in *Quercus variabilis* Seedlings to Environmental Variations Using *In Situ* and *Ex Situ* Experiments

**DOI:** 10.1371/journal.pone.0061192

**Published:** 2013-04-08

**Authors:** Jing-Pin Lei, Wenfa Xiao, Jian-Feng Liu, Dingpeng Xiong, Pengcheng Wang, Lei Pan, Yong Jiang, Mai-He Li

**Affiliations:** 1 Key Laboratory of Tree Breeding and Cultivation of State Forestry Administration, Research Institute of Forestry, Chinese Academy of Forestry, Beijing, People's Republic of China; 2 Research Institute of Forest Ecology, Environment and Protection, Chinese Academy of Forestry, Beijing, People's Republic of China; 3 College of Horticulture and Forestry, Huazhong Agricultural University, Wuhan, China; 4 Hubei Academy of Forestry, Wuhan, People's Republic of China; 5 Swiss Federal Research Institute WSL, Birmensdorf, Switzerland; 6 State Key Laboratory of Forest and Soil Ecology, Institute of Applied Ecology, Chinese Academy of Sciences, Shenyang, People's Republic of China; United States Department of Agriculture, Agricultural Research Service, United States of America

## Abstract

Forest tree species distributed across a wide range of geographical areas are subjected to differential climatic and edaphic conditions and long-term selection, leading to genotypes with morphological and physiological adaptation to the local environment. To test the ability of species to cope with changing environmental conditions, we studied the ecophysiological features of *Quercus variabilis* using seedlings grown in geographically widely isolated populations (Exp. I, *in situ*) and in a common garden (Exp. II, *ex situ*) using seedlings originating from those populations. We found that *Q. variabilis* plants grown in different locations along a south-north gradient had different levels of nutrients (N, P, K) and carbon-physiological performance (photosynthesis, non-structural carbohydrates, such as soluble sugars and starch), and that these physiological differences were not correlated with local soil properties. These geographic variations of plant physiology disappeared when plants from different locations were grown in the same environment. Our results indicate that the physiological performance of *Q. variabilis* plants is mainly determined by the climatic variations across latitude rather than by their soils or by genetic differentiation. The adaptive ability of *Q. variabilis* found in the present study suggests that this species has the potential to cope, at least to some extent, with changing environmental conditions.

## Introduction

The global average temperature has increased by approximately 0.6°C (±0.2°C) over the past 100 years and is projected to continue to rise at a rapid rate [1]. Ecologists typically assume that temperature is a primary fitness determinant of plant growth and survival at high-latitudinal and upper elevational limits [2]–[6]. To predict changes in species' distribution under current and future climate, especially rapid global warming, an understanding of the ecophysiology of plants growing in populations at the northernmost (also uppermost) distribution limit is needed.

Many studies have documented geographic variations in morphology [7]–[9], phenology [10]–[12], ecophysiology [13]–[16], and genetic differentiation [17], [18] among plant populations across geographic ranges. At the ecophysiological level, water use efficiency [19]–[21], stomata [22]–[25], photosynthesis [26]–[28], and nutrients [29]–[31] in plants have been extensively investigated. Villar et al. [32] found that plants grown in regions with sufficient precipitation allocated more biomass to stem and leaves and less to roots. Miyazawa and Lechowicz [33] studied seedlings of 8 north American *Picea* species grown in a common garden and found that the relative growth rate and specific leaf area had a positive relationship with latitude, while leaf size and leaf length were negatively correlated with latitude. Ehleringer and Phillips studied the ecophysiological factors contributing to the distributions of several *Quercus* species and found that leaf size and leaf longevity of *Q. macrocarpa* Michx. and *Q. turbinella* Greene were not correlated with summer water shortage [34].

Nitrogen and phosphorus play vital roles in plant functioning, and are among the most important limiting nutrients in terrestrial ecosystems [35], [36]. Patterns of N, P, and K status in plant tissues, especially in leaves, have been studied intensively [36], [37]. Alpine plants often had a higher leaf N concentration in the polar region than in the equatorial region [38]. Reich and Oleksyn [39] found that leaf N and P concentration increased but N/P ratios decreased with increasing latitude together with decreasing temperature.

Studies indicated that mobile carbohydrate concentration of trees increased with elevation during the growing season [29], [40]–[43], but decreased with increasing elevation up to the alpine treeline during winter [15], [44]–[46]. Unlike with altitude, however, the availability of mobile carbohydrate in plants across broad latitudinal ranges have received little attention. Concentration of mobile carbohydrates reflect the balance between carbon gain (photosynthesis) and loss (structural growth and respiration) [41], [47], [48].

**Figure 1:**
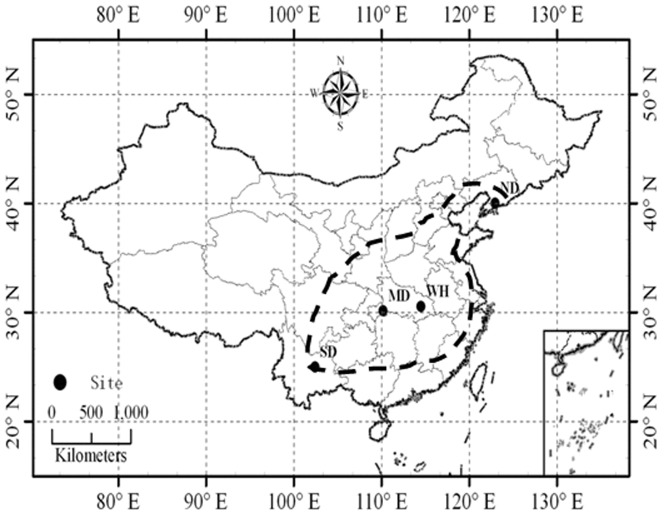
Geographical locations of the study sites in China (Dashed line is the distribution range of *Q. variabilis*) (ND = northern distribution, Zhuang-He in NE China, MD = middle distribution, He-Feng in central China, SD = southern distribution, An-Ning in SW China. WH = Wu-Han in Hubei province, central China).

We studied the ecophysiological characteristics of *Quercus variabilis* Blume using two experiments, i.e. *Q. variabilis* seedlings grown over a latitudinal gradient (Exp. I, *in situ*) and in a common garden (Exp. II, *ex situ*) using seedlings originating from those locations. *Q. variabilis* is geographically widely distributed in China, with the northernmost limit in southern Liaoning Province and the southern boundary in Yunnan Province (Fig. 1). Forest tree species distributed across a wide range of geographical areas are subjected to differential climatic and edaphic conditions and long-term natural selection, leading to generating different genotypes with morphological and physiological adaptation to the local environment. Hence, our hypotheses to be tested are that (1) plants grown in northern populations have higher concentration of nutrients and mobile carbohydrates than those grown in southern populations, to adapt to a relatively harsh environment (e.g. low temperature and short growing season in the north), and (2) the adaptation differences remain when they are grown in other environments.

## Materials and Methods

### Study sites and species

The present study included an *in situ* experiment and an *ex situ* experiment. The object of this study was 3∼5-year-old seedlings of *Q. variabilis* grown in different geographical locations (*in situ*) and in a common garden using seedlings originating from those locations (*ex situ*) (Fig. 1, Table 1). All necessary permits for the described field study were obtained from the local forestry bureaus at the beginning of the experiment. Five naturally generated old growth stands dominated by *Q. variabilis* were selected from its southern distribution (SD, An-Ning in SW China, 102.45°E, 24.99°N), middle distribution (MD, He-Feng in central China, 110.21°E, 30.15°N), and northern distribution area (ND, Zhuang-He in NE China, 122.96°E, 39.99°N) (Fig. 1, Table 1), respectively. Before the growing season of 2009, 3–5 experimental gaps (gap diameter ≈1-fold height of surrounding trees) each with 4–6 naturally generated and healthy seedlings (3–5 years old, 1.0∼1.5 cm in base diameter, and 70∼90 cm in height) were created within each of the five stands in ND, MD, and SD, respectively, so that the seedlings can adapt to similar sun exposure across locations prior to treatment or sampling. Simultaneously, 2–3 randomly selected seedlings out of the 4–6 seedlings within each gap were tagged and remained intact for future sampling (*in situ*), and the other 2–3 seedlings were carefully excavated and transplanted in a common garden (*ex situ*) in the Jiufeng National Forest Park, Wu-Han (WH, 114.91°E, 30.48°N; Fig. 1, Table 1). The seedlings were planted in a randomized complete block design with five blocks (*n* = 5) for seedlings originating from ND, MD, and SD, respectively. Six seedlings (2 rows of 3 plants) were planted at a spacing of 40×40 cm with a margin of 30 cm for each block (100 cm×140 cm with a buffer zone of 50 cm between any two blocks).

**Table 1:** Characteristics of the plots and the sampling sites.

	Elevation m a.s.l.	Slope exposure	MAT (°C)	MAP (mm)	Soil Type	Community
***In situ***						
Northern distribution (ND)	256	SE	7.7	819.6	Brown soil	*Q. variabilies - Pinus koraiensis*
Mid-distribution (MD)	935	SW	13.2	1529.4	Yellow brown soil	*Q. variabilies - Pinus massoniana*
Southern distribution (SD)	1895	SE	14.9	985.8	Red soil	*Q. variabilies - Pinus yunnanensis*
***Ex situ***						
Common garden (WH)	870	SE	17.5	1100.0	Yellow brown soil	

MAT = mean annual temperature; MAP = mean annual precipitation; ND = northern distribution, Zhuang-He in NE China, MD = middle distribution, He-Feng in central China, SD = southern distribution, An-Ning in SW China. WH = Wu-Han in Hubei province, central China.

### Sampling

Samples were taken between August 20 and 28, 2010 (i.e. 2 years after transplanting). Samplings were carried out around noon to minimize the influences of sunlight and temperature on carbohydrate concentration. Each seedling sampled was completely excavated. Leaves (second flush leaves), stem wood (with bark), and fine roots (<0.5 cm in diameter, with bark) were separately collected. A 2-cm long stem segment was taken from the middle part of each stem. Root samples were carefully washed. To obtain a single sample for each tissue for each stand, we mixed the same tissue collected from 5–6 seedlings grown in 3–5 gaps within each stand (*in situ* in ND, MD, and SD, respectively; *n* = 5) or each block (*ex situ*, *n* = 5), in order to reduce the costs of chemical analyses. All samples were immediately stored in an ice box, and killed in a microwave oven within 6 hours, then dried to constant mass at 65°C. Dried plant material was ground to pass a 0.15 mm sieve.

We randomly selected 3 out of the 5 *in situ* stands in ND, MD, and SD, respectively, to take soil samples (*n* = 3). Four to six soil cores each with 3 cm in diameter and 30 cm in depth were taken from the 3–5 gaps within each selected stand, and then pooled to obtain a mixed sample for each stand. All soil samples were air-dried. After removing the stones and plant materials, soils were ground to pass through a 2 mm sieve for chemical analysis.

### Photosynthesis parameters

Photosynthetic photon flux density (PPFD) response curves were made with a portable infrared gas analyzer (Licor 6400, Li-Cor, Lincoln, NE). The assimilation rates were measured on fully expanded leaves from 09:00 to 12:00 h on clear, cloudless days (15–30, August, 2010). The air cuvette temperature, the relative humidity, and the air CO_2_ concentration were maintained at 25±2°C, 50±5%, and 400 µL L^−1^, respectively. PPFD was decreased from 2000 to 0 µmol m^−2^ s^−1^ (2000, 1800, 1600, 1400, 1200, 1000, 800, 600, 400, 200, 100, 80, 50, 20, 0 µmol m^−2^ s^−1^). Assimilation was recorded at each light level following a 5 min acclimation time, and three replications were used for each plant. According to Prioul and Chartier [49], apparent quantum efficiency (AQE), maximum photosynthetic rates (A_max_), dark respiration (R_d_), light compensation points (LCP), and light saturation points (LSP) were calculated from the light response curve data, using the program Photosyn Assistant (Dundee Scientific, Dundee, Scotland).

### Analyses of total soluble sugars and starch

The powdered material (0.1 g) was put into a 10 ml centrifuge tube, where 5 ml of 80% ethanol was added. The mixture was incubated at 80°C in a water bath shaker for 30 min, and then centrifuged at 4000 rpm for 5 min. The pellets were extracted two more times with 80% ethanol. Supernatants were retained, combined and stored at −20°C for soluble sugar determinations. The ethanol-insoluble pellet was used for starch extraction. Glucose was used as a standard. Soluble sugars were determined using the anthrone method [50]. The starch concentration was measured spectrophotometrically at 620 nm using anthrone reagent, and was calculated by multiplying glucose concentrations by the conversion factor of 0.9 [51]. Concentration of sugars and starch was described on a dry matter basis (% d.m.).

### Analyses of plant and soil nutrients

The finely ground plant samples were firstly digested through the Kjeldahl procedure, using H_2_SO_4_ and H_2_O_2_ for digestion, and then the total nitrogen and phosphorus concentrations were determined using the flow injection method, and potassium was determined by applying the flame photometry method [52]. Soil pH was determined by the acidimetry method (soil∶water = 1∶5). Total soil N concentration [11] was measured with the Kjeldahl procedure, total soil P (TP) with Perchloric acid digestion followed by the molybdate colorimetric test, and total soil K (TK) with the flame photometry method. Soil hydrolyzable N (HN) was determined by using alkaline hydrolysis diffusion method, soil available P and K (AP and AK) by Mo-Sb anti-spetrophotography method and the flame photometry method, respectively.

### Data analysis

NSC is defined as the sum of the starch plus the total soluble sugars for each sample. Data (NSC, starch, total soluble sugars, and nutrient concentration) were confirmed for normality by Kolmogorov-Smirnov-Tests. Two-way analysis of variance (ANOVA) was performed for each parameter within each tissue type, using experiments (*in situ* vs. *ex situ*) and origin (ND, MD, and SD) as factors, and found that the responses of most parameters differed with experiments (data not shown). Hence, we analyzed the data for each parameter within each tissue type for each experiment separately, using one-way ANOVA, and followed by multiple comparisons. Pearson's correlation analysis was performed to detect the relationships between physiological parameters and the soil chemical properties across geographic locations where plants grown *in situ*. Differences were considered significant if p<0.05. All statistical analyses were conducted using SPSS 17.0 version (SPSS, Chicago, Illinois, USA).

## Results

### Plant nutrients

#### *In situ* experiment

Seedlings grown in ND had significantly higher tissue N concentration than those grown in MD and SD (p<0.05, Table 2). Nitrogen concentration in leaves and roots of seedlings in ND were 30–39% and 130–188% higher than those in MD and SD, respectively. Tissue P and K concentration did not vary among different geographical locations (Table 2), except that K in roots in MD was 36% and 38% lower than those in ND and SD, respectively.

**Table 2:** Nutrients concentrations (mean ± SD; mg g^−1^, *n* = 5) in *Quercus variabilis* seedlings grown in different geographical locations and in a common garden in China.

Nutrients	Tissues	ND	MD	SD
***In situ***				
N	Leaves	17.17±0.53a	13.16±0.90b	12.36±1.39b
	Stem	5.58±0.51a	4.02±0.52b	2.82±0.32c
	Roots	7.46±0.83a	3.25±0.37b	2.59±0.75b
P	Leaves	1.47±0.37a	1.10±0.23a	1.61±0.18a
	Stem	0.63±0.07a	0.71±0.11 a	0.82±0.07a
	Roots	0.71±0.14 a	0.77±0.58a	1.10±0.21a
K	Leaves	7.38±0.56a	6.72±1.02a	6.27±0.29a
	Stem	3.23±0.19a	3.84±0.65a	3.94±0.42a
	Roots	5.09±0.41a	3.28±0.40b	5.27±0.79a
***Ex situ***				
N	Leaves	14.56±1.36a	13.35±1.87a	13.71±0.71a
	Stem	3.40±0.13a	3.74±0.33a	3.22±0.48a
	Roots	5.47±0.62a	6.07±2.87a	5.19±0.49a
P	Leaves	0.82±0.08a	0.83±0.16a	0.97±0.09a
	Stem	0.31±0.04a	0.36±0.07a	0.68±0.21a
	Roots	0.48±0.05a	0.53±0.32a	1.67±0.62a
K	Leaves	6.35±2.09a	6.66±1.82a	6.96±0.47a
	Stem	2.41±0.71a	3.13±1.23a	4.51±0.72a
	Roots	3.19±0.76a	4.65±1.53a	4.42±0 .93a

Different letters indicate significant difference at p<0.05 level for each row, tested using Duncan's multiple range test. ND = northern distribution, Zhuang-He in NE China, MD = middle distribution, He-Feng in central China, SD = southern distribution, An-Ning in SW China.

#### *Ex situ* experiment

No difference was found in N, P, and K concentration in seedlings grown in the common garden for 2 years after transplanting from different geographical locations (Table 2).

### Photosynthetic responses

#### *In situ* experiment

Seedlings grown in ND, MD and ND showed non-significant difference in AQE (Table 3). SD plants had significantly higher A_max_ compared to plants in ND and MD (p<0.05, Table 3). R_d_ was found to be the smallest in MD plants, while LCP was the least in SD plants (p<0.05, Table 3). LSP did not vary among plants grown in SD, MD, and ND (Table 3).

**Table 3:** Photosynthetic parameters (mean ± SD, *n* = 5) of *Quercus variabilis* seedlings grown in different geographical locations and in a common garden in China.

	AQE	A_max_	R_d_	LCP	LSP
***In situ***					
ND	0.0591±0.0442a	5.2342±1.1017b	2.3938±0.036a	66.1±25.8ab	293.6±78.1a
MD	0.0205±0.0051a	2.5616±1.1463b	0.8901±0.2748b	87.6±4.3a	392.8±35.6a
SD	0.0679±0.0264a	13.1006±2.0737a	1.7349±0.8356ab	29.4±13.3b	504.4±181.4a
***Ex situ***					
ND	0.0338±0.0075a	14.899±0.2402ab	1.6267±0.0756a	61.3±1.5a	637.9±15.3a
MD	0.0353±0.0084a	14.5339±3.2761b	1.8476±0.3845a	68.6±15.1a	659.1±49.9a
SD	0.0443±0.0104a	19.7417±0.7429a	1.9094±0.3087a	53.8±2.6a	714.4±59.7a

ND = northern distribution, Zhuang-He in NE China , MD = middle distribution, He-Feng in central China, SD = southern distribution, An-Ning in SW China; AQE, apparent quantum efficiency, µmol CO_2_/µmol photons; A_max_, maximum photosynthetic rates, µmol m^−2^ s^−1^; R_d_, dark respiration, µmol m^−2^ s^−1^; LCP, light compensation point, µmol m^−2^ s^−1^; LSP, light saturation point, µmol m^−2^ s^−1^. Different letters indicate significant difference at p<0.05 level for each parameter among the three locations within each experiment (i.e. *in situ* or *ex situ*).

#### *Ex situ* experiment

Like plants grown *in situ*, the highest A_max_ was found in plants originating from SD (p<0.05, Table 3). AQE, R_d_, LCP and LSP did not vary among plants originating from ND, MD, and SD in the common garden (Table 3). The statistically significant differences in R_d_ and LCP found in plants grown *in situ* were not found in plants grown *ex situ* (Table 3).

### Responses of mobile carbohydrates

#### *In situ* experiment

Concentration of soluble sugars in stems of SD plants were much less than those in ND and MD plants (p<0.05, Table 4). But roots of MD plants had significantly lower soluble sugar concentration compared to SD and ND plant roots (p<0.05, Table 4). Both leaves and roots of ND plants showed significantly higher starch contents compared to those of MD and SD plants (p<0.05, Table 4). Concentration of NSC in stem and roots were found to be significantly higher in ND plants than in MD and SD plants (p<0.05, Table 4).

**Table 4:** Results of ANOVA analyses for mobile carbohydrates (sugars, starch, NSC) in *Quercus variabilis* seedlings grown in different geographical locations and in a common garden in China.

	*In situ*				*Ex situ*			
	df	F	*P*	Effects	df	F	*P*	Effects
**Soluble sugars**								
Leaves	4	3.605	0.094	No effects	4	4.392	0.067	No effects
Stem	4	12.158	0.008	ND≈MD>SD	4	0.906	0.453	No effects
Roots	4	5.642	0.042	SD≈ND>MD	4	0.619	0.570	No effects
**Starch**								
Leaves	4	79.144	0.000	ND>MD≈SD	4	18.673	0.003	ND≈MD>SD
Stem	4	1.107	0.390	No effects	4	0.346	0.720	No effects
Roots	4	34.153	0.001	ND>MD≈SD	4	3.631	0.093	No effects
**Non-structural carbohydrates (NSC)**								
Leaves	4	1.151	0.378	No effects	4	0.019	0.981	No effects
Stem	4	7.258	0.025	ND>MD≈SD	4	0.792	0.495	No effects
Roots	4	7.441	0.024	ND>MD≈SD	4	1.012	0.418	No effects

ND = northern distribution, Zhuang-He in NE China, MD = middle distribution, He-Feng in central China, SD = southern distribution, An-Ning in SW China.

#### *Ex situ* experiment

Two years after transplanting seedlings into the common garden, concentration of mobile carbohydrates in tissues did not differ among plants originating from ND, MD, and SD (Table 4), except for the starch concentration in leaves of plants originating from SD which was significantly lower than that in plants originating from ND (increased by +61%) and MD (+72%) (p<0.05, Table 4).

### Allocation of nutrients and carbohydrates within the plant

#### *In situ* experiment

Only P allocation to roots and K allocation to leaves differed significantly among ND, MD, and SD plants grown *in situ* (p<0.05, Table 5). From north to south, plants invested more P into roots, but less K into leaves (Table 5). The allocation of soluble sugars and NSC to stem decreased but to roots increased in plants grown *in situ* from north to south (Table 5).

**Table 5:** Allocation (mean % ± SD, *n* = 5) of nutrients and mobile carbohydrates within a *Quercus variabilis* seedling grown in different geographical locations and in a common garden in China.

	*In situ*			*Ex situ*		
	ND	MD	SD	ND	MD	SD
**N**						
Leaves	0.35±0.06	0.35±0.09	0.38±0.07	0.36±0.02	0.30±0.06	0.40±0.10
Stem	0.22±0.03	0.20±0.13	0.18±0.06	0.23±0.03a	0.18±0.06ab	0.13±0.01b
Roots	0.43±0.07	0.45±0.06	0.44±0.11	0.41±0.05	0.52±0.11	0.48±0.11
**P**						
Leaves	0.31±0.09	0.21±0.10	0.17±0.04	0.26±0.01a	0.24±0.07a	0.14±0.03b
Stem	0.26±0.05	0.20±0.05	0.18±0.06	0.28±0.05a	0.21±0.05ab	0.13±0.02b
Roots	0.43±0.10b	0.59±0.06ab	0.65±0.10a	0.46±0.06b	0.55±0.12ab	0.73±0.06a
**K**						
Leaves	0.26±0.05a	0.21±0.04ab	0.15±0.02b	0.28±0.03	0.22±0.03	0.26±0.07
Stem	0.22±0.03	0.23±0.18	0.18±0.05	0.30±0.10	0.20±0.01	0.23±0.02
Roots	0.51±0.07	0.55±0.14	0.67±0.07	0.42±0.08b	0.58±0.04a	0.51±0.08ab
**Soluble sugars**						
Leaves	0.16±0.03	0.15±0.02	0.12±0.05	0.17±0.05	0.14±0.02	0.22±0.09
Stem	0.24±0.03a	0.15±0.10ab	0.10±0.03b	0.26±0.03a	0.15±0.05b	0.13±0.01b
Roots	0.60±0.06b	0.70±0.08ab	0.78±0.07a	0.57±0.08	0.71±0.05	0.64±0.10
**Starch**						
Leaves	0.09±0.02a	0.03±0.01b	0.04±0.02b	0.15±0.07	0.16±0.03	0.08±0.05
Stem	0.19±0.02	0.14±0.09	0.20±0.02	0.12±0.01a	0.08±0.02b	0.09±0.03ab
Roots	0.72±0.03	0.83±0.09	0.76±0.03	0.73±0.06	0.77±0.04	0.82±0.02
**Non-structural carbohydrates (NSC)**						
Leaves	0.15±0.03	0.13±0.01	0.11±0.04	0.17±0.05	0.15±0.02	0.21±0.09
Stem	0.23±0.02a	0.15±0.10ab	0.11±0.02b	0.24±0.02a	0.13±0.04b	0.13±0.01b
Roots	0.62±0.05b	0.72±0.08ab	0.78±0.07a	0.60±0.08	0.72±0.04	0.67±0.09

ND = northern distribution, Zhuang-He in NE China; MD = middle distribution, He-Feng in central China; SD = southern distribution; An-Ning in SW China; No letters indicate non-significant difference, and different letters indicate significant difference at p<0.05 level for each parameter in each tissue type among the three locations within each experiment (i.e. *in situ* or *ex situ*).

#### *Ex situ* experiment

Plants originating from the north tended to allocate more N and P to stem, as well as more P to leaves, but less P and K to roots compared to plants originating from the south (Table 5). Differences in allocation of mobile carbohydrates were detected only for stem in plants originating from different locations grown in the common garden, showing a decreased trend for mobile carbohydrates (sugars, starch, and NSC) from ND, to MD and SD plants (Table 5).

### Relationship between physiological parameters and soil nutrients

**Figure 2:**
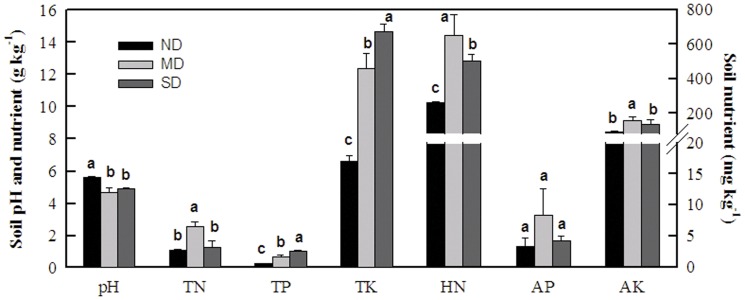
Soil pH and nutrients in different geographical locations (ND = northern distribution, Zhuang-He in NE China, MD = middle distribution , He-Feng in central China, SD = southern distribution, An-Ning in SW China). TN, TP, and TK were total N, P, and K contents in g kg^−1^ soil (+1 SD), respectively. HN, AP, and AK were hydrolyzable N, available P and K in mg kg^−1^ soil (+1 SD), respectively. Different letters indicate significant difference (p<0.05) within each parameter among the three locations.

Soils in the 3 populations *in situ* were acid soil with pH values ranging from 4.7 to 5.6 (Fig. 2). MD showed higher concentration of total N, hydrolyzable N, and available P and K (Fig. 2). Results of Pearson's correlation analysis indicated that plant nutrients, photosynthetic parameters and mobile carbohydrates all were not correlated with soil nutrients for *Q. variabilis* grown across scales *in situ* (data not shown).

## Discussion

### Plant nutrients

Geographic locations significantly affected N but not P and K concentration in *Q. variabilies* plants (*in situ*, Table 2). Discrepancy of nutrient concentration in a plant species or functional type across large distribution range has been observed [53]–[56]. Reich and Oleksyn [39] found that leaf N and P concentration declined towards the equator as the average temperature and the growing season length increase. A meta-analysis with 753 terrestrial plant species in China found that leaf N and P concentration increased with increasing latitude [57]. Similarly, leaf N, P and K of *Q. liaotungensis* Koidz. [58] and *Celtis australis* L. [59] were found to increase with the increase of elevation (i.e. decrease of temperature). However, Kerkhoff et al. [60] reported that leaf N and P were not correlated with latitude.

Different *Q. variabilies* provenances grown under the same conditions did not show any differences in nutrient concentration (*ex situ*, Table 2). This may imply that the nutrient concentration of *Q. variabilies* is mainly determined by its growing environment. The same climate conditions (temperature and precipitation) and soil nutrient availability led to similar concentration of nutrients in tissues (*ex situ*, Table 2). However, previous studies of *Pinus sylvestris* L.found that N and P concentration in needles were higher [56], [61], and K concentration were lower in northern provenance than in southern provenances grown in a common garden [62]. Leaf N of *Populus trichocarpa* Torr. & A. Gray ex Hook. was also found to be higher in northern provenance than in southern provenance in a common garden [63].

Patterns of nutrient allocation did not differ among ND, MD, and SD *Q. variabilis* plants, except for northern plants invested less P to roots but more K to leaves compared to southern plants (*in situ*, Table 5). Domisch et al. [64] found that soil temperature did not affect the allocation patterns of N or P between shoots and roots in *P. sylvestris* seedlings. But Xu et al. [65] found that higher temperature induced *Populus cathayana* Rehd. cuttings to allocate more N to the aboveground organs.

Previous studies indicated that leaf N increased with increasing latitude as a result of decreasing mean annual temperature [39], [56], [57], [66], and our results gained from plants grown *in situ* (Table 2) were consistent with this. Temperature-related plant physiological stoichiometry and cold temperature effects on biogeochemistry associated with soil nutrient supply may contribute to such trend [39], [67]. The results from Weih and Karlsson [67] suggested that increased leaf N concentration with increasing latitude and/or altitude was not only a passive consequence of weaker N dilution by declined growth rate, but also a physiological acclimation to lower growth temperature. Hence, it may also be possible that such a trend is resulted from the adaptation strategy of plants to their growing conditions, reflecting the metabolic adaptation of leaves producing more protein to acclimate to the cold environment, because N is integral to proteins involved in photosynthesis process.

### Photosynthesis and non-structural carbohydrates

*Q. variabilis* plants grown both *in situ* and *ex situ* showed significantly higher assimilation rates in southern than in northern plants (Table 3). Other *in situ* experiments indicated that the maximum photosynthetic rate was highest in plants grown in the middle part of the distribution area for *P. sylvestris* [68] and *Eucryphia cordifolia* Cav. [69], and decreased northwards and southwards. Significant increases in photosynthesis rate were found in red alder (*Alnus rubra* Bong.) grown along a geographic gradient from southeast to northwest in China [70]. A common garden experiment using *Clarkia unguiculata* Lindl. plants from 16 populations across latitudes found that the maximum photosynthesis rates decreased with increasing latitude of plant origin [71]. However, photosynthetic rate was found to increase with increasing latitude of origin in five provenances of black cottonwood [63]. Such increasing trends of photosynthesis were also observed in other species, e.g. for *Populus balsamifera* L. [72], [73], *Picea abies* (L.) Karst. [74], *Alnus sinuate* (Regel) Rydb. and *Betula papyrifera* Marsh. [75] grown in a common garden. The populations/provenances from locations with lower temperature and shorter growing season had higher maximum photosynthetic rates, which may reflect plants' adaptation to produce more carbohydrates within the short growing season.

No differences in leaf dark respiration were found in different provenances of *Q. variabilis* grown in the common garden (Table 3). In line with our finding, previous common garden studies also showed little evidence for differences in leaf dark respiration rates in geographically contrasting sources of *Pinus taeda* L. [76], *P. banksiana* Lamb. [77], *Quercus alba* L., *Q. rubra* L.[78], *Acer rubrum* L. [78], [79], and *A. saccharum* Marsh. [80].

*Q. variabilis* plants grown in north tended to have higher concentrations of mobile carbohydrates (NSC, sugars, and starch) than plants grown in south *in situ* (Table 4). But when plants originating from different geographic locations grown in the common garden, those differences disappeared except for starch in leaves (Table 4). However, *P. sylvestris* seedlings [81] and *Alcantarea imperialis* Rubra plants [82] were found to have higher concentration levels of mobile carbohydrates under higher soil temperature compared to lower temperature. Oleksyn et al. [83] found that total non-structural carbohydrate concentrations were significantly higher in roots and needles of *P. sylvestris* originating from 50° than 60°N. But for the same species, it was also reported that concentration of mobile carbohydrates decreased in needles but increased in roots with latitude of origin [84].

More than 60% of the mobile carbohydrates (sugars, starch, NSC) were invested into roots, and south plants allocated more carbohydrates to roots than north plants did (Table 5). The percentage of carbohydrates stored in roots gained in the present study was consistent with the results reported by Canham et al. [85]. Allocation pattern of carbohydrates was found to be affected by temperature (e.g. along elevational or latitudinal gradients) [81], [82], [86], and nutrients available [87]–[89]. The present study found that the north plants allocated more NSC to the stem but less NSC to the roots compared to the south plants (Table 5).

The lack of clear relationships between plant physiological parameters and soil nutrients across scales found in the present study may suggest that climate discrepancy is the major contributor to the differences in physiology of *Q. variabilis* plants growing in different geographic populations. Although soil nutrients are essential for plant growth, there was no correlation between the supply of nutrients and the concentration of mineral nutrients in plant tissues, indicating that plant nutrition may be mainly determined by plants' absorption and utilization rather than the pool size of nutrients in soil [90], [91].

### Conclusion

Today's plant communities are the result of long-term adaptation to their growth environment including climatic impacts. Plant distribution is largely determined by climatic conditions [4], [92]. Plant species distributed across a wide range of environmental conditions, may differentiate genetically, leading to generating ecotypes with different functional traits. Inconsistent with our hypotheses, the differences in nutrient and carbon physiology found among plants grown across geographic locations disappeared when they were transplanted to grow in the same environment (Tables 2, 3, 4). Our results showed that the physiological performance of *Q. variabilis* plants may be mainly determined by the climate variations across scales but not by different soil conditions, indicating that this species has a high degree of plasticity and is highly flexible in terms of its physiology, and can adapt readily to a range of sites. This adaptation ability of *Q. variabilis* found in the present study suggests that *Q. variabilis* has the potential to cope, at least to some extent, with changing environmental conditions, as proposed recently by Zhu et al. [16] and Li et al. [93] for other *Quercus* species facing to climate changes.
